# Critical Analysis of Assessment Studies of the Animal Ethics Review Process ^†^

**DOI:** 10.3390/ani3030907

**Published:** 2013-09-04

**Authors:** Orsolya Varga

**Affiliations:** Laboratory Animal Science Group, IBMC (Instituto de Biologia Molecular e Celular), Universidade do Porto, Rua do Campo Alegre 823, 4150-180 Porto, Portugal; E-Mail: ovarga@ibmc.up.pt; Tel.: +351-226-074-900; Fax: +351-226-099-157

**Keywords:** Animal Ethics Committees, outcome assessment, benefits

## Abstract

**Simple Summary:**

In many countries, the approval of animal research projects depends on the decisions of the ethics committees which review the projects. Since the efficiency of the protection of experimental animals greatly depends on the performance of the ethics committees, its regular assessment is crucial. This paper reviews the results of studies assessing the performance of the ethics committees, and emphasizes the importance of outcome assessment in the evaluation of the performance of ethics committees.

**Abstract:**

In many countries the approval of animal research projects depends on the decisions of Animal Ethics Committees (AEC’s), which review the projects. An animal ethics review is performed as part of the authorization process and therefore performed routinely, but comprehensive information about how well the review system works is not available. This paper reviews studies that assess the performance of animal ethics committees by using Donabedian’s structure-process-outcome model. The paper points out that it is well recognised that AECs differ in structure, in their decision-making methods, in the time they take to review proposals and that they also make inconsistent decisions. On the other hand, we know little about the quality of outcomes, and to what extent decisions have been incorporated into daily scientific activity, and we know almost nothing about how well AECs work from the animal protection point of view. In order to emphasise this viewpoint in the assessment of AECs, the paper provides an example of measures for outcome assessment. The animal suffering is considered as a potential measure for outcome assessment of the ethics review. Although this approach has limitations, outcome assessment would significantly increase our understanding of the performance of AECs.

## 1. Introduction

The purpose of the present study is twofold: to critically review studies that assess the performance of animal ethics committees and to provide an example to illustrate how outcome assessment, which is the most neglected among assessment studies, could be useful in this area.

In many countries, in particular in Europe, North America and Australasia the approval of animal research projects depends on the decisions of committees which review the projects. These entities which will be referred to here as AEC’s, are given the task to protect the welfare of animals and to ensure that animals are used in a way that is scientifically worthwhile.

Animal ethics committees were established worldwide as part of the legal protection of research animals, which lays down the rules for the structure of these committees and define the main goals for their functioning [1,2]. Besides the main function of authorizing research project, there are no harmonized standards for functions, leading to significant variety between AECs. Some play a role in education of committee members [3], others perform retrospective severity assessment [4] or giving advice to scientists [5]. This variability, at least in the EU, may soon be reduced, since the new Directive 2010/63/EU on the protection of animals used for scientific purposes was approved on 22nd September 2010 and entered into force in Member States on 1st January 2013 hopefully leading to a more harmonized animal protection in the European Union. The new Directive aims to enforce conducting experiments which are designed to cause the least pain, suffering, distress or lasting harm. Compared to the previous Directive 86/609, a number of new requirements have appeared, including the need for procedures to be assigned a severity classification prospectively and the actual severity experienced by each animal during the course of a procedure to be determined and reported retrospectively. However, the most important change is that all scientific procedures have to be conducted under a project authorization approved by the national competent authorities.

Animal ethics review is performed as part of the authorization process and therefore performed routinely, but comprehensive information about how well the review system works is not available. 

Thus, in this paper, first I looked at available information on animal ethics review and structured it to fit Donabedian’s model. Donabedian has proposed that performance in research and healthcare can be assessed by evaluating the structure, the process and the outcome [6] as presented in Table 1. His model has later been implemented for assessment of other services such as education [7]. 

In the second part of the paper, I explored why it is challenging to address the question whether AECs achieved their objectives, and finally, I provided an example of measures for outcome assessment, without which efficacy of AECs cannot be fully evaluated. 

**Table 1:** Donabedian’ structure-process-outcome model (adapted from [8] about the three types of administrative evaluations).

Component to be evaluated	Evaluation approach	Explanation
Resource input	Evaluation of Structure	Structure assessment relates to the organization of ethics committees, the number, training and competency of staff, the comprehensiveness of services, and the accessibility of services. The link to outcome assessment is based on the assumption that the better the structure, the better the process and outcomes.
Service rendered	Evaluation of Process	This addresses issues related to what committees do, how they do it and the interaction they have with each other/researchers.
Outcome achieved	Evaluation of Outcome	Outcome assessment relate specifically to the program’s objectives. Narrow and measurable objectives are needed.

## 2. “Assessment Studies” in the Literature

In order to find empirical studies which addressed assessment of AECs I searched Academic Search Complete (keyword combination: “ANIMAL experimentation” AND “committee” AND “welfare”; number of citations retrieved is 73) and Google Scholar (keyword combination: “interview” OR “survey” OR “observation” AND “decision making” AND “committee” AND “ethics” AND "animal experiment"; number of citations retrieved is 135) until 2012. Additionally, further studies which were identified by citations in retrieved papers have been added (number of citations retrieved is 17). After removing duplications 223 references were selected in a systematic manner, based on the information in the title, abstract or in the full text-paper. Papers were excluded if (1) instead of AEC’s they focused on scientific, philosophical or other topics (n = 139), (2) if they were not based on empirical methods (n = 14), (3) publication type was not of interest (n = 50) or (4) were not published in English (n = 1). The remaining 19 papers, on which this review is based on, were grouped according to Donabedian’s model (Table 2). 

**Table 2:** Assessment studies, structured according to Donabedian’s model.

	Goals	References
Evaluation of Structure	Evaluation of committee composition and dynamics, recruitment of members, workload, participation level and member turnover	[9,10,11,12,13]
	Evaluation of members’ opinion on structure, organization function and performance	
Evaluation of Process	Attitude of committee members towards ethics review	[13,14,15,16]
	Decision making process (individual and group)	[17,18,19]
	Policy implementation and variation in time for review	[19,20,21]
	Examination of variation among AECs in evaluation of hypothetical or real cases	[22,23,24]
Evaluation of Outcome	Compliance of investigators	[25]
	Approval rate	[21,26,27]

The selected empirical studies are diverse in their methodology; they used interviews with committee members, questionnaires, observational studies, reviews of written documents and protocol evaluations (details of the methodologies used in each paper are listed in Table 3).

Findings of these assessment studies are shortly presented below, structured according to Donabedian’s model. Note, that some of the studies fit into more than one category. 

### 2.1. Evaluation of Structure

A study, prepared by a non-governmental organization of health care professionals promoting alternatives to animal research, interviewing a panel of former members of Institutional Animal Care and Use Committees (IACUCs) concluded—among other problems—that the *composition of the committees* is not balanced, that scientists are over-represented and that this influences the performance of the committee [11]. Besides its methodological weaknesses the study missed to clarify basic definitions (e.g., “good performance”), although the aim of this study was to identify ways to improve IACUC performance. 

Similar issues were investigated by better designed studies, such as the Canadian work that examined in detail how the effectiveness of an AEC was influenced by committee composition and dynamics, recruitment of members, workload, participation level and member turnover. The effectiveness was defined in the paper as achieving the mandate of the committee to protect research subjects. This includes meeting procedural standards of committee independence, broad expertise, sufficient depth of review, commitment of members to the mandate, and fair and respectful committee discussion. In this study 28 members of AECs at four universities in western Canada were interviewed. A bias towards institutional or scientific interests was found and the authors also concluded that protocol review may be influenced by heavy workloads, type of review process and lack of full committee participation [9]. Very similar observations were made in a study that analyzed the overall membership of IACUCs at leading U.S. research institutions. Committees and their leadership are comprised of a preponderance of animal researchers, and other members who are affiliated with each institution; some of whom also work in animal laboratories. The study concluded that this composition leads to bias in favour of approving animal experiments and reduce the overall objectivity and effectiveness of the oversight system [10].

A United States based study investigated the committee members’ *opinion on the structure and organizational functions of IACUCs*. It has to be noted that IACUCs are self regulated, although using various protocols. This study found that in the eyes of IACUC members, their committees are generally promoting the welfare of laboratory animals and complying with applicable federal regulations. Most respondents believed a single, institution-based IACUC was an appropriate venue for institutional approval of animal care and use, that their IACUCs represented their institutions' constituencies and that the unaffiliated IACUC members adequately represented their surrounding communities. However, opinions of IACUC members differed significantly from those of unaffiliated IACUC members [12]. In the second part of the study the authors carried out a self-assessment survey on IACUC function and effectiveness. They found that 98% of all respondents believed that their IACUCs advanced animal welfare, but in many instances, veterinarians’ responses to individual survey items were significantly different from those of other IACUC members [13].

### 2.2. Evaluation of Process

This category is broken down into four parts: studies that addressed attitude of committee members towards ethics review, studies on decision-making process, studies on how law is implemented by AECs, and studies on inconsistency between decisions at individual and group level.

*Attitude of committee members towards ethics review* was examined by several studies. Canadian AEC members were interviewed on various ethical matters, including ethics, animal ethics, science and ethics, and the use of animals in research, in order to explore their implicit ethical framework. The results revealed that AEC members hold quite a narrow view on both animal ethics and animal use in research, and that they apply implicit ethical notions, such as respect and justice, when performing ethical evaluations of animal use [14]. Ideland confirmed (through interviews with Swedish AEC members) that the different personal views on what ethics means, and hierarchies among committee members, characterise the meetings. However, committee traditions and priorities of interpretation were also considered important to the decision [16]. Graham (2002) made similar findings in a smaller study in 2002, investigating the attitude of US committee members towards the assessment of scientific merit and the use of alternatives in research proposals [15]. The study revealed that it was not clear for the committee members what they should assess in the ethics review. 

*Decision-making process* has been examined at group and individual levels; both processes are important for the consistency of the ethical decisions. A recent Canadian study aimed to understand how committee members make decisions and how effective they are in implementing policy and achieving their stated aims. The primary finding was that the focus of protocol review by committee members was reducing harm to animals, with less focus on the ethical justification of research despite this being stressed in policy as a goal of AECs. The author also believes that AEC effectiveness could be improved by clarifying the elements of harm-benefit assessment and the relation between AEC and scientific peer review [19]. Another Canadian observational study focusing on the ethical issues debated during the ethical review process revealed that the majority of comments were technical. However, the ethical concerns were implicit in both scientific and technical language, or some of the scientific and technical comments had an impact on the ethical treatment of animals [17]. The only study which partly aimed to understand how ‘carefully’ AECs assessed *animal suffering* of GM animals, analysed applications submitted to animal ethics committees in Sweden during 2002. The study revealed that applications were often approved by the committees despite containing insufficient information regarding ethically relevant aspects, and that the arguments for using GM animals were often unclear [18].

A German study investigated how *legal changes affect the performance* of ethical committees. Using questionnaires addressed to licensing authorities and members of ethics committees, Kolar and Ruhdel found that the inclusion of animal welfare into the German constitution did not change or changed only to a small extent the decisions [21]. A study which addressed several issues, aimed to evaluate impact of the *legal process* called “just-in-time” (JIT) on the IACUC which is an optional process that allows for submission of a grant proposal with funding dependent on subsequent verification of IACUC approval. The new process seemed to be less successful than was expected. 59% of respondents indicated that they experienced no reduction in workload. Of those who indicated a reduction, the amount of reduction varied from “little” to 40% [20]. 

*Inconsistencies* between decisions made by different committees were reported by two studies. Dresser (1989) had 32 institutional AECs in the US reviewing 4 hypothetic protocols involving experimental procedures frequently conducted on animals to check for reliability of decisions [22]. Committees were in general in agreement on the need to refine the protocols to minimize pain, distress, and other harm to animals, but there was less agreement in the approach to assessing the justification for laboratory animal use. Apparently, this component of committee responsibilities presented major conceptual and practical difficulties for committees engaged in animal research review [22]. These results are corroborated in Plous and Herzog’s 2001 study, in which 50 US AEC’s re-evaluated three real, randomly selected cases, previously evaluated by one of the committees. No significant relation was found between the original committee evaluation and the re-evaluation by Plous and Herzog. Since it was speculated that difference in final authorization is caused by procedural difference between committees, individual evaluations were assessed regarding several dimensions of evaluation. Significant disagreement was found in all aspects except for the one where detailed classification criteria were given (expected animal pain). In a workshop setting, Voipo confirmed that there is a large variation between individuals in scoring the degree of costs, benefits and the possibilities of modifying costs, e.g., by introducing an improved or refined technique that is less distressing to the animal [24].

### 2.3. Evaluation of Outcome

In the surprisingly few studies aiming to assess outcomes, two approaches have been used: one used a statistical approach to provide numbers of applications granted, declined or suggested for modifications; the other approach focused on effectiveness and researchers’ compliance—*i.e.*, to what extent researchers experimenting on animals follow ethical decisions. 

(1) Hau *et al.* investigated the minutes (reports) of Swedish AEC meetings held between 1989 and 2000 (in a total of 3,607 meetings) to find out about approval rates [26,27]. A great majority of the applications received were approved. However, 18.1% of them were approved only after modifications. When the applications for experimental work in animals that resulted in requests for modification were further analyzed, it was found that the majority of the changes requested could be classified as ‘Refinement’. The results suggest that the work of the committees may be perceived as an ongoing process, since several of the applications for which modification was requested were projects that had been approved on a previous occasion but were now up for renewal [26,27]. Some argue that the high approval rate shows that AECs do not function well e.g., [19] however, others argued that there is no standard for what proportion of proposals should be rejected [14]. 

(2) The survey based study by Ingham aimed to measure the successfulness of IACUC by considering investigator compliance and finding ways to improve that. The consultant who was hired developed a questionnaire by using information obtained from confidential interviews held with IACUC members and key animal-users at the facility. That part of the survey which focused on the review process reported general satisfaction of researchers but Ingham noted—without details—overly negative comments as well. The direct impact of the study was that it allowed specific actions to be taken to improve overall IACUC effectiveness immediately such as changes in the software package used for completing the Animal Procedure Statement in 2000 [25].

### 2.4. Summary of the Different Evaluation Studies

Although animal experiments are regularly performed all over world and their regulation involves AECs, very few empirical studies exist that evaluate the function of these entities. What is more, the identified assessment studies have been provided by few groups and the studies had specific research questions and were also limited in geographical sense. Detailed background information on the authors and each included article is presented in Table 3. 

**Table 3:** Background information of each included article.

Authors (Year) Title [citation number]		
	**Affiliation**	
	**Funding**	**Conflict of interest**
	**Method**	
Dresser, R., (1989) Developing standards in animal research review [22]		
	School of Law, Case Western Reserve University, Cleveland, USA	
	Not specified	Not specified
	Protocol evaluation, protocol review form analysis	
Graham, K. (2002) A study of three IACUCs and their views of scientific merit and alternatives [15]		
	Center for Animals and Public Policy, Tufts University School of Veterinary Medicine, USA	
	Not specified	Not specified
	Survey and interview	
Hau, J., Carlsson, H. E. Hagelin, J. (2001) Animal research. Ethics committees have influenced animal experiments in Sweden [27]		
	Division of Comparative Medicine, Department of Physiology Uppsala University, Uppsala, Sweden	
	Not specified	Not specified
	Review of minutes of meetings	
Hagelin, J., Hau, J. and Carlsson, H. E. (2003) The refining influence of ethics committees on animal experimentation in Sweden [26]		
	Department of Physiology, Division of Comparative Medicine, Uppsala University, Sweden.	
	Helge Axson Johnsons Stiftelse, Magn. Bergwalls Stiftelse, CF Lundstroms Stiftelse and CFN (grant. no. 02-72)	Not specified
	Review of minutes of meetings	
Hansen, L. A., (1), Goodman, J. R. (2), Chandna, A. (3) (2012) Analysis of Animal Research Ethics Committee Membership at American Institutions [10]		
	1 Departments of Neurosciences and Pathology, University of California San Diego, USA; 2 People for the Ethical Treatment of Animals, Washington, DC 20036, USA; 3 Department of Sociology and Criminal Justice, Marymount University, USA	
	Not specified	none
	Membership review	
Houde, L., Dumas, C. (1) Leroux, T. (2), (2003) Animal ethical evaluation: an observational study of Canadian IACUCs [17]		
	1 Département de psychologie, Université du Québec à Montréal, Montréal, QC, Canada, H3C 3P8.; 2 Centre de recherche en droit public Université de Montréal	
	Not specified	Not specified
	Observation	
Houde, L., Dumas, C. (1) Leroux, T. (2), (2009) Ethics: views from IACUC members [14]		
	1 Département de Psychologie, Université du Québec à Montréal, Canada; 2 Centre de Recherche en Droit; Public, Université de Montréal, Canada	
	Not specified	Not specified
	Interview	
Ideland, M. (2009) Different views on ethics: how animal ethics is situated in a committee culture [16]		
	School of Teacher Education, Malmö University, Malmö, Sweden.	
	Not specified	Not specified
	Interview	
Ingham, K. M., Klein, H. J., Kastello, M. D. (1), Goldberg, J. A., (2) Johnson, R. G. (3), (2000) A novel approach for assessing the quality and effectiveness of IACUC oversight in investigator compliance [25]		
	1 Department of Laboratory Animal Resources, Merck Research Laboratories, USA 2 Management Development Systems, Inc., Hillsborough, NJ, USA 3 Chiron Vaccines, Emeryville, CA, USA	
	Not specified	Not specified
	Interview and survey	
Kolar, R. and Ruhdel, I. (2007) A survey concerning the work of ethics committees and licensing authorities for animal experiments in Germany [21]		
	Animal Welfare Academy (Akademie für Tierschutz), German Animal Welfare Federation (Deutscher Tierschutzbund), Neubiberg, Germany	
	Not specified	Not specified
	Survey	
Mann, M. D. , Prentice, E. D., (2007) Verification of IACUC approval and the just-in-time PHS grant process [20]		
	University of Nebraska Medical Center, Omaha, NE, USA.	
	Not specified	Not specified
	Survey	
Nordgren, A. (1) Röcklinsberg, H. (2), (2005) Genetically modified animals in research: an analysis of applications submitted to ethics committees on animal experimentation in Sweden [18]		
	1 Centre for Applied Ethics, Linköping University, SE–581 83 Linköping, Sweden; 2 Centre for Theology and Religious Studies, Lund University, Allhelgona Kyrkogata 8, SE–223 62 Lund, Sweden.	
	Swedish Foundation for Strategic Research (the ELSA Program) and the Knut and Alice Wallenberg Foundation (Program of ethics research in connection to Swegene and WCN).	Not specified
	Protocol evaluation	
Plous, S. (1) Herzog, H. (2), (2001) Animal research. Reliability of protocol reviews for animal research [23]		
	1 Department of Psychology, Wesleyan University, Middletown, CT 06459-0408, USA. 2 Department of Psychology, Western Carolina University, Cullowhee, NC 28723, USA.	
	Not specified	Not specified
	Protocol evaluation	
Schuppli, Catherine A., (2011) Decisions about the Use of Animals in Research: Ethical Reflection by Animal Ethics Committee Members [19]		
	Animal Welfare Program, Faculty of Land and Food Systems, University of British Columbia, Canada	
	International Foundation for Ethical Research (IFER) through a postgraduate fellowship; by the UBC Animal Welfare Program that is funded by the Natural Sciences and Engineering Research Council of Canada; the BCSPCA; the BC Veterinary Medical Association; and sponsors listed on the programme website	Not specified
	Observation and interview	
Schuppli, C. A. and Fraser, D., (2007) Factors influencing the effectiveness of research ethics committees [9]		
	Animal Welfare Program, Faculty of Land and Food Systems, University of British Columbia, Vancouver, British Columbia, Canada	
	The International Foundation for Ethical Research (IFER) through a postgraduate fellowship; by the UBC Animal Welfare Program that is funded by the Natural Sciences and Engineering Research Council of Canada; the BCSPCA; the BC Veterinary Medical Association; and other sponsors listed on the programme website at www.landfood.ubc.ca/animalwelfare/.	none
	Interview	
Silverman, J. (1), Baker, S. P. (2), Lidz, C. W. (3), (2012) A self-assessment survey of the Institutional Animal Care and Use Committee, part 2: structure and organizational functions [12]		
	1 Department of Animal Medicine, University of Massachusetts Medical School, Worcester, MA. 2 Departments of Quantitative Health Sciences, Cell Biology, and Information Services, University of Massachusetts Medical School, Worcester, MA. 3 Department of Psychiatry, University of Massachusetts Medical School, Worcester, MA.; USA	
	Not specified	none
	Survey	
Silverman, J. (1), Baker, S. P. (2), Lidz, C. W. (3), (2012) A self-assessment survey of the Institutional Animal Care and Use Committee, Part 1: animal welfare and protocol compliance [13]		
	1 Department of Animal Medicine, University of Massachusetts Medical School, Worcester, MA, USA 2 Departments of Quantitative Health Sciences, Cell Biology, and Information Services, University of Massachusetts Medical School, Worcester, MA, USA 3 Department of Psychiatry, University of Massachusetts Medical School, Worcester, MA, USA	
	Not specified	none
	Survey	
Voipio, H.-M., (1), Kaliste, E., Nevalainen, T. (2), Hirsjärvi, P.,(3) Ritskes-Hoitinga, M.(4), (2004) Nordic-European Workshop on Ethical Evaluation of Animal Experiments [24]		
	1 Laboratory Animal Centre, University of Oulu, Oulu, Finland; 2 National Laboratory Animal Center, University of Kuopio, Kuopio, Finland; 3 Department of Social and Moral Philosophy, University of Helsinki, Helsinki, Finland; 4 Biomedical Laboratory, University of Southern Denmark, Odense C, Denmark	
	The workshop was organized by the Cooperation Group for Laboratory Animal Sciences of the Finnish Ministry of Education and made possible through funding by the Academy of Finland, the Finnish Ministry of Education and Agriculture and Forestry, NOVA University and the Finnish Society for the Protection of Animals.	Not specified
	Protocol evaluation	
Physicians Committee for Responsible Medicine (PCRM), (1994) Animal Care and Use Committees: Structural Problems Impair Usefulness [11]		
	Physicians Committee for Responsible Medicine (PCRM), USA	
	Not specified	Not specified
	Interview	

The key findings of the studies are that AECs differ in structure, in their decision-making methods, in the time they take to review proposals and that they also make inconsistent decisions. These findings are consistent with international reports [3,28]. There are clearly too few studies dedicated to the quality of outcomes, and to what extent decisions have been incorporated into daily scientific activity. The few existing ones showed that AECs do not incorporate legal changes into daily decisions and individual ethical decisions may ignore ethically important aspects. According to Donabedian’s model full quality assessment—in this particular case of the efficacy of AECs—cannot be performed without proper evaluation of all three aspects (structure, process and outcomes). Thus, there is a clear need for further and more thorough outcome evaluation of AECs.

## 3. Why is it Challenging to Study the Outcome of AECs’ Decisions?

In theory, efficacy of ethics committees could be determined by checking whether they achieve their aim, *i.e.*, to make right (ethical) decisions. Although this looks simple, in reality it is a very challenging task. Probably for the very same reason, lack of evaluation of efficacy was also identified as a problem, when the human research ethics committees (RECs) were evaluated [29]. A recent systematic review of empirical data on evaluation of human research ethics committees found that of 43 studies published in the US, none of them addressed efficacy analysis [30]. As potential reasons, there are both (i) theoretical (what is the right decision) and (ii) methodological (how to identify the right decisions) difficulties with efficacy assessment. 

(i) Ethical justification is a complex issue and committees do not have access to a single moral truth, to which their judgment is supposed to correspond [31]. In fact, the complexity of ethical issues and the diversity of views in society are primary reasons why decisions are made by committees composed by a diverse group of people. As Prentice added it may not be desirable to make committee decisions more uniform, but structural and process interventions may help to improve the quality of decision-making [32].

(ii) There is no objective tool which could be used to select morally acceptable animal experimentation. Using harm-benefit analysis is a common way of ethical assessment but it is far from being objective. 

However, even if the morality of ethical decisions cannot be assessed it is possible to identify sets of measures which may be used to describe the performance of ethics committees. For example, in an early study on outcome assessment of clinical ethics committees the primary outcomes were intensive care unit days, hospital days, and life-sustaining treatments in those patients who did not survive to hospital discharge. It was assumed in the study that ethics consultation would serve to reduce intensive care unit days in those patients who would not have survived to hospital discharge, but would have no effect on this outcome among those who did survive [33]. 

The core methodological problem of outcome assessment of AECs is that impacts are complex including effect on animals’ wellbeing, experiments quality, and researchers’ attitude towards animal research. The impacts are interconnected and not easily separable, ‘clear’ causality can hardly be studied. AECs’ ethical decisions have direct impact on how experiments were carried out. However, if the compliance of the researchers carrying out the particular research is not sufficient, the improvements asked by AECs will not necessarily be apparent on animal wellbeing. Thus, animal wellbeing as a simple outcome measure may not truly reflect the efficacy of AECs decisions. 

### 3.1. An Example of Measures for Outcome Assessment

Selecting appropriate outcome measures for assessing the efficacy of AECs is far from straightforward. 

During ethics review AECs have to pursue numerous objectives established by law which may vary from country to country and there is a strong *focus on the three Rs* (Replacement, Reduction and Refinement). For example, the (research) animal protection laws of six countries or regions with the highest animal use (United States, Japan, China, European Union, Australia, Canada) [34] incorporate the 3Rs into the ethical review. 

The variety of objectives of research animal protection laws of the above six countries are presented in Table 4. 

Considering that the animal research legislation has been introduced and developed with the aim of protecting animals used in research, well functioning legislation is expected to result in reduced animal suffering. It is also appropriate to focus outcome assessment on this measure given the great moral importance attributed to animal welfare. In their original publication on the 3Rs, Russell and Burch placed great emphasis on minimising pain and distress to the individual animal. The outstanding importance of refinement was also articulated by the Swiss Academy of Medical Sciences which laid down that “If the suffering of individual animals can be reduced significantly through the use of a larger number of animals, the *reduction of individual suffering* shall take priority over the reduction of the number of animals used in the experiment.” [35].

**Table 4:** Objectives of legislation regulating animal research use with references.

Principles	Objectives	Sources
Applying 3Rs	Reducing number of experimental animals keeping scientific value of research	[4,5,36,37,38,39,40]
	Reducing animals’ suffering by refinement such as anaesthesia, humane endpoints, euthanasia	
	Replacing animals to non animal tools and use of less sensitive animals	
Severity assessment	Recognizing severity of procedures	[4,5,38,40]
Scientific relevance	Reviewing the merit and value of research under review	[4,5,38,39]
	Maximizing benefit	
Ethical justification	Ensuring that the benefits of research outweighs the risks (harm-benefit analysis)	[4,5,38,39]
	Giving extra protection to certain species such as non-human primate research	
	Preventing duplication of experiments	
Expertise of researchers	Well trained personal is involved in experiments	[4,5,38,39,40]
Appropriate design	Methodological rigour of the research is necessary	[4,5,38,39,40]
	Statistical soundness	
	Selected species can be scientifically justified	

Thus, I assume that the most relevant objective of ethics review is to reduce the harm inflicted to animals. The question is whether actual suffering of individual animals can serve as appropriate measures of the efficacy of animal ethics review. Regarding animal welfare studies, pain (suffering) that animals experience can be recognized and quantified [41]. At the point of ethics review, experiments are classified in terms of expected severity, *i.e.*, how much harm they are expected to cause to animals. These systems typically have 3–5 classes, ranging from no or little impact to severe effects. 

The severity classification system now being introduced in the EU relating to pain and suffering experienced by animals used in research has three grades, research projects are classified as ‘mild, ‘moderate’, ‘severe’. Additional category is the ‘non-recovery’ which means the animal is anesthetized and killed without recovering consciousness. This system is presently being introduced into the legislation of the 27 member states of the EU with the new Directive on the protection of animals used for experimental and other scientific purposes (2010/63 EU). The directive requires monitoring research animals’ wellbeing, quantifying data on actual suffering and reporting it to the competent authorities in the respective member states. It is too early to say how well this system will actually work, and whether it will result in a wider and more uniform assessment of harm caused to animals in research. If successful, this will be useful in terms of producing data on animal suffering which could allow evaluating to what extent AECs contribute to the reduction of animals’ suffering by comparing the expected and actual suffering of animals. 

Although the possibility of evaluation of AECs’ performance from the animals’ point of view is already a step forward, the proposed approach has clear drawbacks. First, performing the assessment implies significant workload. Second, it will have an inherent bias towards severe experiments. A comparison of predicted and retrospectively evaluated harm will only be possible for projects that are under retrospective evaluation (e.g., projects in which non-human primates are used (Art. 16 of 2010/63 EU), projects where the procedures on the experimental animals were classified as severe (Art. 54 of 2010/63 EU), reuse of experimental animals is proposed). Third, it may also be debated to which extent this comparison actually reflects the functioning of the ethics review system in particular and not the effect of legislation as a whole. 

## 4. Conclusions

By using Donabedian’s model, dividing AEC-evaluation studies into structure-, process- and outcome-assessment works, I have shown that existing empirical studies mainly addressed structural and process questions, while outcome was overlooked. The efficient operation of AECs, however is crucial for biomedical research, thus full assessment of their work is of utmost importance and cannot be done without equal consideration of their structure, process and outcome. 

The main difficulty with outcome assessment seems to be that there is no consensus definition for outcomes of AECs decisions. When do they work well? If they make legal decisions, or if their decisions are followed (compliance) or are they functioning well if their decisions prevent unnecessary harm caused to animals? Presently, the few studies dealing with outcome assessment focused mostly on statistical analysis of approval, while investigating compliance and the ‘correctness of decisions’ is unduly ‘neglected’. In the light of the common deception, “ranging from omitting information to outright lying in research”, and information on clinical researchers perspectives on value of ethics review [42], further studies are necessary to understand to what extent ethical decisions are followed [43]. The aim is not to point out non-compliance of animal researchers but to contribute to increase legitimacy of AECs by facilitating communication between researchers and AECs [44].

Although outcome assessment studies focusing on efficacy of AECs should be more regularly performed, they are challenging to carry out. In order to contribute to the improvement in outcome assessment, in this paper I have suggested animal suffering as a potential measure for outcome assessment of ethics review. The introduction of a uniform system for assessing severity of animal experiments across the EU (Directive 2010/63/EU on the protection of animals used for scientific purposes), despite being challenging, will potentially allow a comparison of data on expected suffering and actual suffering, which may provide a measure of AEC performance. 
